# Association between body mass index, metabolic syndrome and common urologic conditions: a cross-sectional study using a large multi-institutional database from the United States

**DOI:** 10.1080/07853890.2023.2197293

**Published:** 2023-04-10

**Authors:** Maria Camila Suarez Arbelaez, Sirpi Nackeeran, Khushi Shah, Ruben Blachman-Braun, Isaac Bronson, Maxwell Towe, Abhishek Bhat, Robert Marcovich, Ranjith Ramasamy, Hemendra N. Shah

**Affiliations:** a Desai Sethi Urology Institute, University of Miami Miller School of Medicine, Miami, FL, USA; b UMass Chann Medical School, University of Massachusetts, Amherst, MA, USA

**Keywords:** BMI**;** diabetes mellitus, erectile dysfunction, lithiasis, metabolic syndrome, obesity, overactive bladder, prostate cancer

## Abstract

**Introduction:**

The study aims to determine whether body mass index (BMI), metabolic syndrome (MS) or its individual components (primary hypertension, type 2 diabetes mellitus and dyslipidemias) are risk factors for common urological diseases.

**Materials and methods:**

Cross-sectional study with data collected on February 28, 2022 from the TriNetX Research Network. Patients were divided in cohorts according to their BMI, presence of MS (BMI > 30 kg/m^2^, type 2 diabetes mellitus, primary hypertension and disorders of lipoprotein metabolism) and its individual components and its association with common urological conditions was determined. For each analysis, odds ratio (OR) with 95% confidence intervals were calculated. Statistical significance was assessed at *p* < .05.

**Results:**

BMI > 30 kg/m^2^ was associated with increased risk of lithiasis, kidney cancer, overactive bladder, male hypogonadism, benign prostatic hyperplasia, and erectile dysfunction (*p* < .05). On the contrary, BMI was inversely associated with ureteral, bladder and prostate cancer (*p* < .05). In all urological diseases, MS was the strongest risk factor, with prostate cancer (OR = 2.53) showing the weakest and male hypogonadism the strongest (OR = 13.00) associations.

**Conclusions:**

MS and its individual components were significant risk factors for common urological conditions. Hence holistic approaches with lifestyle modification might prevent common urological disease.

**Key messages**
- Overall, metabolic syndrome is the strongest risk factor for all the analysed urological diseases.
- Abnormally high body mass index can be a risk or protective factor depending on the threshold and urological disease that are being evaluated.
- Metabolic syndrome and increased BMI should be considered important factors associated to the prevalence of common urological diseases.

## Introduction

Obesity and metabolic syndrome (MS) are now recognized as global epidemics [1,2]. According to the 2017–2018 National Health and Nutrition Examination Survey, 42.5% of United States (U.S.) adults aged 20 and over have obesity, as defined by body mass index (BMI) ≥ 30.0, and another 31.1% are overweighted (BMI 25.0–29.9) [3]. In addition, a positive trend in the incidence of MS has been seen from 2007 to 2012, with the prevalence during this period being approximately 34.2% [4]. Obesity and MS represent a serious public health issue, not only because they increase the risk of mortality and morbidity [1,5], but also because the medical spending attributable to these pathologies ranges from $78.5 billion for obesity [6] to trillions of dollars for MS [1]. It is estimated that the total healthcare costs due to obesity is likely to double every decade, and projections calculate that it will represent 18% of total US healthcare costs by 2030 [7].

Urologic diseases such as kidney and upper tract urothelial cancers [8], urolithiasis [9], benign prostatic hyperplasia (BPH) [10], erectile dysfunction (ED) [11], among others, have also been increasing annually. Although the aetiology of these diseases is likely multifactorial, underlying obesity and MS could potentially be impacting their incidence. Although several studies have explored the impact of obesity and MS on urologic diseases [2, 12], the majority of them have evaluated either a single urologic pathology [13–15] or the study population did not surpass the thousands [16,17]. Moreover, some published studies provide contradictory results [18–20].

Given this background, we analysed TriNetX Research Network data, which contains information about more than 80 million patients across 49 healthcare organizations (HCOs) from the U.S. The primary aim of this study was to determine whether obesity and MS are risk factors for common urologic diseases such as prostate cancer, ureteral cancer, kidney cancer, bladder cancer, kidney stones, BPH, hypogonadism, ED and overactive bladder (OAB). Secondarily, we aim to analyse the risk of the above-mentioned diseases depending on the BMI-category and for each of the MS components – hypertension, diabetes mellitus (DM) and dyslipidaemia. As obesity and MS are preventable diseases, we believe that knowing their impact on urologic health is relevant not only for physicians but also for patients’ holistic care [21].

## Methods

### Data source

To determine associations between chronic health conditions and urologic conditions, we used the TriNetX Research Network from July 2015 to February 2022 [18]. TriNetX only reports on population level data without including protected health information identifiers. The deidentified dataset al.so does not provide data on individual hospitals. As study procedures on this database involve only the analysis of deidentified data, and it is not possible to readily identify the individuals about whom the data were collected, the projects analysing this database may not involve ‘human subjects’ and therefore not require individual IRB approval [19,20]. Special privacy measures were taken during data acquisition, and any filter that resulted in less than or equal to 10 patients would reveal only a numerical value of 10. Certain filters and codes were also restricted for this reason. The authors had access to TriNetX database that was supported by an educational grant from ACERUS Pharmaceuticals. ACERUS Pharmaceuticals was not involved in the planning, design, writing or any other aspect of this project.

### Variables and outcome definitions

We assessed the potential associations between BMI thresholds and MS with urologic conditions, using the following diagnoses and ICD-10 codes: type 2 DM (E11), overweight and obesity (E66), essential (primary) hypertension (I10) and disorders of lipoprotein metabolism and other lipidaemia (E78). We additionally determined the percentage of each cohort that had potentially complicated comorbid conditions, including ischaemic heart diseases (I20-25), alcohol related disorders (F10) and nicotine dependence (F17). We determined associations with urologic conditions if they had a diagnosis code for any of the following diseases: stones (N20-23), ureteral cancer (C66), kidney cancer (C64), bladder cancer (C67), OAB (N32.81), prostate cancer (C61), male hypogonadism (E29.1), male ED (N52) and BPH (N40).

### Cohorts and statistical analysis

To measure associations of BMI with urologic conditions, we constructed cohorts using the following BMI thresholds: <25 kg/m^2^, <30 kg/m^2^, >30 kg/m^2^, >35 kg/m^2^, >40 kg/m^2^. Comparisons were made between all BMI groups against the <25 kg/m^2^ group, which is considered the normal value [22]. The MS cohort included only patients diagnosed with all of the following: type 2 DM (E11), overweight and obesity (E66), essential (primary) hypertension (I10) and disorders of lipoprotein metabolism and other lipidaemia (E78)). This cohort was then compared against a control group labelled No MS, which lacked any of those diagnoses. To better understand the effects of individual MS diagnoses on associations with urologic conditions, we then constructed sub-cohorts of patients with BMI >30 kg/m^2^ and made the following comparisons: type 2 DM vs no type 2 DM, essential hypertension vs no essential hypertension and hyperlipidaemia vs no hyperlipidaemia.

For each analysis, we reported the number of patients with each outcome in each cohort and calculated the Odds Ratio (OR) with 95% confidence intervals. We then used greedy nearest neighbour 1:1 propensity score matching to create ‘balanced’ cohorts and recalculated the ORs. The propensity score matched through the TriNetX platform limits the number of variables that can be used to propensity score match by sample size. For this reason, the propensity score matched for the maximum number of variables that was allowed by the sample sizes for each analysis. We used age, race, ethnicity, DM, hypertension and/or hyperlipidaemia whenever possible or appropriate as detailed in each table. All categorical variables were compared using the chi-squared test and all continuous variables were compared using the t-test with significance assessed at *p*<.05.

## Results

A total of 36,911,824 subjects were included in the study of which 32.5% had obesity (BMI≥ 30 kg/m^2^) and 2.7% had MS. The baseline characteristics of each cohort are elaborated in Table 1. All differences observed had statistical significance (*p* < .05). We found that groups with higher BMI tended to be older, more predominantly female and more likely to have DM, hypertension and hyperlipidaemia. We additionally found that patients with MS were far more likely to have ischaemic heart disease (42% vs 2%), alcohol related disorders (5% vs 1%) and nicotine dependence (20% vs 4%) than the corresponding control group (*p* < .05 in all).

**Table 1.** Baseline characteristics of all the analysed cohorts.

Variable	BMI < 25 (kg/m^2^)	BMI < 30 (kg/m^2^)	BMI > 30 (kg/m^2^)	BMI > 35 (kg/m^2^)	BMI > 40 (kg/m^2^)	No metabolic syndrome	Metabolic syndrome
No. in cohort	9,516,366	15,399,671	7,137,799	3,314,462	1,543,526	32,059,747	1,011,728
Age (years)	37 + 26	43 + 25	51 + 18	50 + 18	49 + 17	40 + 23	65 + 13
Gender							
Male	45%	53%	42%	36%	32%	45%	46%
Female	55%	47%	57%	63%	67%	55%	53%
Race							
White	60%	62%	62%	61%	59%	58%	67%
Black	15%	14%	18%	21%	23%	11%	21%
Asian	4%	3%	1%	1%	1%	3%	1%
Native	<1%	1%	1%	1%	1%	1%	<1%
American							
Unknown	21%	20%	18%	16%	16%	27%	11%
Ethnicity							
Hispanic	6%	7%	8%	7%	7%	9%	9%
Not Hispanic	65%	66%	69%	72%	71%	52%	67%
Unknown	29%	27%	23%	21%	22%	39%	24%
Hypertension	19%	24%	38%	39%	41%	0%	100%
Type 2 Diabetes	7%	8%	19%	22%	24%	0%	100%
Hyperlipidaemia	15%	19%	27%	27%	27%	0%	100%
Ischaemic heart disease	8%	10%	11%	10%	10%	2%	42%
Smoking history	11%	11%	12%	11%	12%	4%	20%
Alcohol use	4%	3%	3%	2%	2%	1%	5%

**Figure 1.**
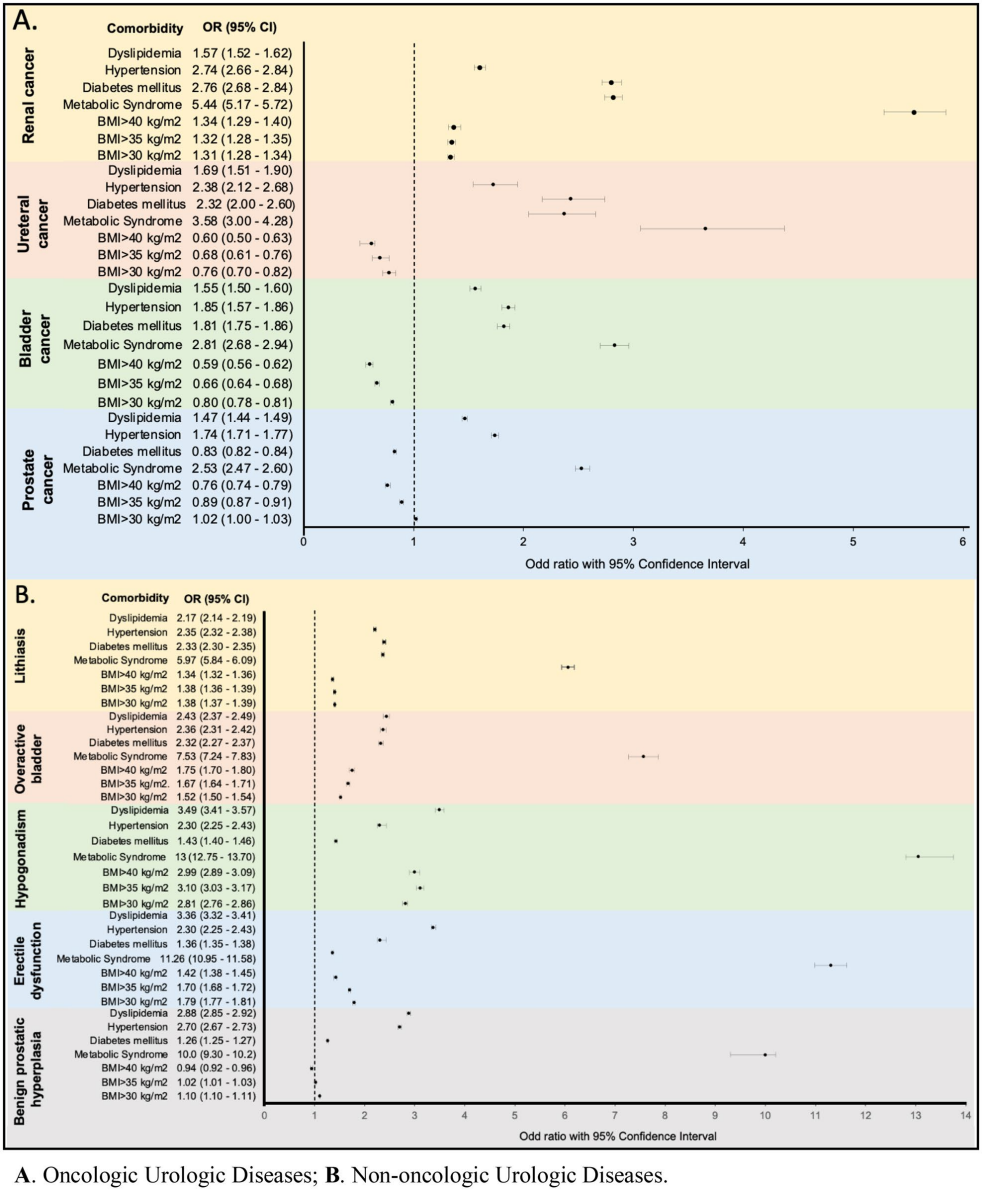
Forest plot of the odds ratio of metabolic syndrome and body mass index in urologic diseases.

Following propensity score matching by age, race and ethnicity, we found that when comparing all the different thresholds of obesity (BMI ≥30 kg/m^2^) to a normal BMI (≤25 kg/m^2^), obesity was associated with significantly increased risk of urolithiasis, kidney cancer, OAB, male hypogonadism, BPH and ED. (Table 2, Figure 1). On the contrary, BMI had inverse associations with ureteral, bladder and prostate cancer. Across all other comparisons between BMI thresholds, directionality of associations remained consistent, except for prostate cancer and BPH in the comparison between BMI >30 kg/m^2^ and BMI >35 kg/m^2^ groups against the BMI < 25 kg/m^2^ group, in which the diseases had decreased associations with higher BMIs (Table 2).

**Table 2.** Measures of association of common urologic diseases with different obesity BMI-thresholds.

Disease	*N* BMI < 25	BMI < 25 vs BMI > 30			BMI < 25 vs BMI > 35			BMI < 25 vs BMI > 40		
		*N* BMI > 30	Unbalanced OR (95% CI)	Balanced OR (95% CI)	*N* BMI > 35	Unbalanced OR (95% CI)	Balanced OR (95% CI)	*N* BMI > 40	Unbalanced OR (95% CI)	Balanced OR (95% CI)
Urolithiasis	129,626	209,241	2.19 (2.17–2.20)	1.38 (1.37–1.39)	98,523	2.22 (2.20–2.24)	1.38 (1.36–1.39)	44,141	2.13 (2.11–2.15)	1.34 (1.32–1.36)
Ureteral cancer	1743	1449	1.11 (1.03–1.19)	0.76 (0.70–0.82)	557	0.92 (0.83–1.01)	0.68 (0.61–0.76)	180	0.63 (0.55–0.74)	0.60 (0.50–0.73)
Kidney cancer	17,044	28,859	2.03 (1.99–2.07)	1.31 (1.28–1.34)	12,140	2.05 (2.00–2.10)	1.32 (1.28–1.35)	5332	1.93 (1.87–1.99)	1.34 (1.29–1.40)
Bladder cancer	22,243	19,308	1.16 (1.14–1.18)	0.80 (0.78–0.81)	6924	0.89 (0.87–0.92)	0.66 (0.64–0.68)	2386	0.66 (0.63–0.69)	0.59 (0.56–0.62)
Prostate cancer	51,172	60,083	1.79 (1.77–1.81)	1.02 (1.00–1.03)	18,940	1.40 (1.38–1.43)	0.89 (0.97–0.91)	5501	0.98 (0.95–1.01)	0.76 (0.74–0.79)
Overactive bladder	64,928	49,530	2.18 (2.15–2.21)	1.52 (1.50–1.54)	26,950	2.39 (2.35–2.43)	1.67 (1.64–1.71)	12,862	2.45 (2.40–2.50)	1.75 (1.70–1.80)
Male hypogonadism	17,656	69,020	5.70 (5.60–5.79)	2.81 (2.76–2.86)	31,449	6.50 (6.38–6.62)	3.10 (3.03–3.17)	12,873	6.44 (6.30–6.59)	2.99 (2.89–3.09)
Erectile dysfunction	57,182	152,948	3.98 (3.94–4.01)	1.79 (1.77–1.81)	58,932	3.81 (3.77–3.86)	1.70 (1.68–1.72)	20,456	3.18 (3.13–3.23)	1.42 (1.39–1.45)
BPH	155,142	205,320	1.96 (1.94–1.97)	1.10 (1.10–1.11)	72,623	1.71 (1.69–1.73)	1.02 (1.01–1.03)	24,354	1.37 (1.36–1.39)	0.94 (0.92–0.96)

*Notes: N* = number of patients; propensity score matched by age, race, ethnicity; prior to matching, BMI < 25 *N* = 9,511,236; BMI > 30 *N* = 7,135,035; BMI > 35 *N* = 3,314,462; BMI > 40 *N* = 1,543,526; male hypogonadism, benign prostatic hyperplasia and erectile dysfunction were calculated only in males (BMI > 40 *N* = 494,080; BMI > 35 *N* = 1,196,555; BMI > 30 *N* = 2,841,383; BMI < 25 *N* = 4,265,626). *p*<.05 for all comparison except where *p*= .75.

After propensity score matching, we found that all urologic diseases had an increased association with MS (Table 3). The directionality of all associations remained consistent with sub-cohort analyses of DM, hypertension and hyperlipidaemia, except for prostate cancer within the DM analysis, which became inversely associated (OR 0.83, 95% CI 0.82–0.84, *p*<.0001) (Table 4).

**Table 3.** Measures of association between metabolic syndrome and no metabolic syndrome.

Disease	Metabolic syndrome *N*	No metabolic syndrome N	Unbalanced OR (95% CI)	Balanced OR (95% CI)
Urolithiasis	60,815		7.55 (7.48–7.62)	5.97 (5.84–6.09)
Kidney cancer	9577	22,463	13.12 (12.81–13.44)	5.44 (5.17–5.72)
Bladder cancer	7152	25,647	8.56 (8.34–8.79)	2.81 (2.68–2.94)
Overactive bladder	21,065	46,731	14.03 (13.80–14.26)	7.53 (7.24–7.83)
Prostate cancer	22,769	125,108	9.00 (8.87–9.13)	2.53 (2.47–2.60)
Male hypogonadism	19,359	53,048	18.36 (18.05–18.67)	13.00 (12.35–13.70)
Erectile dysfunction	54,448	132,057	22.45 (22.22–22.69)	11.26 (10.95–11.58)
Benign prostatic hyperplasia	90,832	180,343	30.06 (29.81–30.32)	10.00 (9.80–10.20)

*Notes: N* = number of patients; propensity score matched by age; prior to matching, metabolic syndrome *N* = 1,011,239; no metabolic syndrome *N* = 32,040,979; male hypogonadism, benign prostatic hyperplasia and erectile dysfunction were calculated only in males (metabolic syndrome *N* = 466,011; no metabolic syndrome *N* = 23,998,575). *p*<.0001 for all comparison.

**Table 4.** Measure of association between metabolic syndrome components and urology diseases.

	Diabetes mellitus type 2^b^				Hypertension^c^				Dyslipidaemia^d^			
Disease	Yes	No	Unbalanced OR (95% CI)	Balanced OR (95% CI)	Yes	No	Unbalanced OR (95% CI)	Balanced OR (95% CI)	Yes	No	Unbalanced OR (95% CI)	Balanced OR (95% CI)
Lithiasis	81,543	174,485	2.14 (2.13–2.16)	2.33 (2.30–2.35)	155,775	94,464	2.83 (2.81–2.85)	2.35 (2.32–2.38)	131,208	118,065	2.70 (2.68–2.72)	2.17 (2.14–2.19)
Ureteral cancer	839	^a^1399	2.67 (2.46–2.91)	2.32 (2.07–2.60)	1728	476	6.03 (5.45–6.68)	2.38 (2.12–2.68)	1400	794	4.15 (3.80–4.53)	1.69 (1.51–1.90)
Kidney cancer	13,471	20,007	3.02 (2.95–3.09)	2.76 (2.68–2.84)	25,447	7,798	5.46 (5.32–5.60)	2.74 (2.66–2.83)	19,701	13,334	3.50 (3.42–3.57)	1.57 (1.52–1.62)
Bladder cancer	11,088	18,190	2.73 (2.67–2.80)	1.81 (1.75–1.86)	21,628	7,317	4.94 (4.81–5.07)	1.85 (1.79–1.91)	18,223	10,638	4.05 (3.96–4.15)	1.55 (1.50–1.60)
Overactive bladder	23,364	43,345	2.42 (2.39–2.46)	2.32 (2.27–2.37)	46,752	18,856	4.17 (4.10–4.24)	2.36 (2.31–2.42)	41,969	23,030	4.34 (4.27–4.41)	2.43 (2.37–2.49)
Prostate cancer	29,514	62,244	1.88 (1.85–1.91)	0.83 (0.82–0.84)	65,590	23,052	3.70 (3.65–3.76)	1.74 (1.71–1.77)	57,417	32,990	3.40 (3.35–3.44)	1.47 (1.44–1.49)
Male hypogonadism	25,546	55,208	1.83 (1.80–1.86)	1.43 (1.40–1.46)	52,214	27,142	2.69 (2.65–2.73)	2.30 (2.25–2.43)	51,150	27,231	3.66 (3.60–3.71)	3.49 (3.41–3.57)
Erectile dysfunction	69,780	126,201	2.27 (2.25–2.29)	1.36 (1.35–1.38)	140,948	52,314	3.96 (3.92–4.00)	2.56 (2.53–2.60)	133,560	56,546	4.87 (4.82–4.92)	3.36 (3.32–3.41)
BPH	118,654	172,650	2.99 (2.97–3.02)	1.26 (1.25–1.27)	231,255	55,584	6.53 (6.47–6.60)	2.70 (2.67–2.73)	213,574	69,935	6.74 (6.68–6.80)	2.88 (2.85–2.92)

*Notes:* Male hypogonadism, benign prostatic hyperplasia and erectile dysfunction were calculated only in males (type 2 diabetes mellitus *N* = 740,996; no type 2 diabetes mellitus *N* = 2,884,093; essential hypertension *N* = 1,529,491; no essential hypertension *N* = 2,094,572; dyslipidaemia *N* = 1,254,746; no dyslipidaemia *N* = 2,369,317); *p*<.0001 for all comparison.

^a^Propensity score matched by age, race, ethnicity.

^b^Propensity score matched by age, hyperlipidaemia and hypertension.

^c^Propensity score matched by age, hyperlipidaemia and diabetes mellitus.

^d^Propensity score matched by age, diabetes mellitus and hypertension.

## Discussion

Our analysis of a large database including more than 80 million patients across 49 HCOs from the U.S., reported that MS increases the risk of all common urologic disease, and obesity is associated to most of them.

### Overactive bladder

Our results show that as BMI increases, the odds of OAB become higher (Figure 1). Some studies reported that one unit increase in BMI caused a 5% increase in the risk of incontinence [23]. Similarly, a five-unit increase in BMI was associated with a 30% increased risk of severe incontinence [24]. An increase in intra-abdominal and intravesical pressure has been proposed as the mechanisms causing heightened risk of OAB in patients with obesity [25].

We found that not only MS (OR = 7.5), but also each of its components, when analysed individually (obesity OR = 1.33, DM OR = 2.32, hypertension OR = 2.36 and dyslipidaemia OR = 2.43), were associated with increased odds of OAB. Ströher et al. found that an increased waist circumference, triglyceride levels, and glycaemia were more prevalent in women with OAB than in women without OAB [26]. Similarly, data from the first and second Nurses’ Health Studies and from HERS, showed a significant correlation between DM and OAB [27,28]. It is proposed that MS induces oxidative stress and profibrotic activity in the bladder which thereby results in OAB [29].

### Kidney cancer

Obesity and hypertension are well known risk factors for renal cancer [30,31], and our results are concordant with these findings. It has been proposed that for each 1 kg/m increase in BMI, the risk of renal cancer increases by 1.06 [32]. In our results, we observed a weakly positive trend of increased renal cell cancer risk with increasing BMI. In the Chinese Kailuan Male Cohort Study, overweight, hypertension, dyslipidaemia, and MS were identified as potential risk factors of renal cell carcinoma [33]. Although we found each of these conditions to exert an increase in the risk of renal cancer, of the MS components, DM was associated with the highest risk of renal cancer (OR 2.76). There is controversy surrounding this topic, as some studies have not been able to establish a relationship between renal cancer and DM, and the association has only been found in the female population [30,34,35]. It is thus possible that the increased risk of renal cancer due to DM in our study could be secondary to the fact that most of our population were women. Although the mechanisms underlying the association between renal cancer, obesity and MS have not been well elucidated, there is a proven role of obesity and MS in the response to treatment and life-expectancy in patients with renal cancer [36,37]. Further studies are required to better characterize the underlying pathophysiology among this association.

### Urolithiasis

Obesity and being overweight have been associated with an increased risk of kidney lithiasis formation as body fatness stimulates the traffic of lithogenic substances, such as uric acid and oxalate [38,39]. The relative risk of kidney stone formation in men and women with BMI> 30 kg/m2 has been reported to be 1.33 and 2.09, respectively [40]. We found similar results in our cohort, in which the odds of kidney lithiasis were increased in patients with a highly abnormal BMI. It has been proposed that the amount of visceral adipose tissue is what increases the risk of urolithiasis [41] in people with high BMIs.

We found that MS increases the risk of urolithiasis by 5.97. Large cohort studies have reported that individuals with MS are twice as likely to have nephrolithiasis than individuals without MS^36^. Similarly, a cross-sectional analysis, using Third National Health and Nutrition Examination Survey data, proposed that the more components an individual had of MS the higher the incidence of self-reported urolithiasis [42]. In our study, we found that hypertension, DM and dyslipidaemia were independently associated with an increased risk of lithiasis, similar to findings reported in the literature [43–45]. Low urine pH and impaired urinary excretion of ammonium seen in MS have been proposed as the mechanism responsible for an increase in urolithiasis formation [46].

### Male hypogonadism

In our study, the OR of the diagnosis of male hypogonadism with a BMI > 30 kg/m^2^ was 1.79 and with BMI > 35 kg/m^2^ was 3.10. Obesity and testosterone deficiency have been described as a vicious cycle [47]. The state of hyperestrogenism seen in obesity decreases the levels of follicle-stimulating hormone and testosterone, all in proportion to the degree of obesity, causing hypogonadotropic hypogonadism [48]. Subsequently, low androgen levels impair lipid metabolism and promotes visceral fat accumulation [49]. Furthermore, MS has been closely associated with hypogonadism [12]. Our results indicated that from all the urologic disease included in the study, hypogonadism presented the highest increase in risk due to MS (OR 13.00; *p*<.05).

In addition, we observed that all the analysed components of MS, when evaluated independently, increased the risk of hypogonadism, with dyslipidaemia having the highest OR (3.49; *p*<.05). Adipocytokines, insulin and blood pressure have been shown to have an inverse association with androgen levels [50–52], explaining why testosterone replacement reduces glycated haemoglobin, visceral adiposity and total cholesterol [52].

### Erectile dysfunction

The main cause of ED pathogenesis is endothelial dysfunction, which can be secondary to other causes such as atherosclerosis [53]. Obesity, hypertension, DM and hyperlipidaemia are risk factors for cardiovascular disease [54], and subsequently, for ED. Our results could support this assertion, as we found all the evaluated components of MS to increase the risk of ED. Dyslipidaemia was the factor with the strongest association (OR 3.36; *p*<.05), a finding that has also been reported in the study by Eaton et al. in which patients with ED had 2.1 times the odds of having elevated total cholesterol/high-density lipoprotein ratio.

### Prostate cancer

The role of MS and its components in the incidence of prostate cancer is controversial. We found that a BMI > 30 kg/m^2^ is associated with an increased risk of prostate cancer (OR 1.79, *p*<.05). Similarly, Andersson et al. found that a higher BMI was not only associated with increasing incidence of prostate cancer but was even more strongly associated with prostate cancer specific mortality [55]. A meta-analysis, including 68,753 participants, demonstrated an overall 5% increase in prostate cancer risk per 5 kg/m2 increased increment in BMI [56]. Nonetheless, we observed that the association between a high BMI and an increase in the risk of prostate cancer was lost when patients had a BMI > 35 kg/m^2^ (OR 0.89; *p*<.05) and a BMI > 40 kg/m^2^ (OR 0.76; *p*<.05). There is even a large prospective cohort study, conducted in 8922 men, in which no association was established between body weight and prostate cancer [13]. The discrepancy in results may be explained by the fact that carcinogenesis implies an abnormal metabolic rate in which an involuntary decrease in skeletal mass occurs [57], making it difficult to identify a potential association between BMI and any cancer. Our results also suggested that MS is associated with prostate cancer (OR 2.53; *p*<.05). Laukkanen et al. also found that the presence of MS increased the risk of prostate cancer to 1.9-fold after adjustment for age, alcohol consumption, physical fitness and diet [58].

However, when we individually evaluated each component of MS, only hypertension and dyslipidaemia were associated with an increased risk of prostate cancer. For instance, it has been reported that hypertension can increase the risk of prostate cancer up to 15% [59], and that each 12-mm Hg increase in diastolic blood pressure could increase the incidence of prostate cancer by 8% [60]. On the contrary, DM has been shown to reduce the risk of prostate cancer. In a multivariate analysis, diabetes was related to a 17% reduced risk for total prostate cancer and a 31% reduced risk for high-grade prostate cancer [61]. We found that the OR of diabetes in the incidence of prostate cancer was 0.83 (*p*<.05). Further investigation is needed to better understand the why the different components of MS exert distinctive associations with prostate cancer.

The possible relation between MS and prostate cancer has been proposed to be an increase in sympathetic activity, which drives prostate gland growth [62], and a dysregulation in the production of specific cytokines, IGF-1, which disrupts the prostate’s normal functioning [63].

### Ureteral cancer

This is the first study in which an association between MS and ureteral cancer has been evaluated. We found that MS is associated with an increased risk of ureteral cancer (OR 3.58; *p*<.05). Nevertheless, when we analysed each component of MS separately, obesity was not associated with an increased incidence of ureteral cancer (OR 0.76; *p*<.05). However, DM, hypertension and dyslipidaemia did show a statistically significant positive association with ureteral cancer (OR 2.32; OR 2.38 and OR 1.69, respectively, with *p*<.05 in all). Further studies should be done to support these findings and their reproducibility.

### Bladder cancer

Controversy surrounds the role of obesity and MS in the incidence of bladder cancer. Several studies have proposed a linear relationship between BMI and the incidence of bladder cancer, suggesting that incremental increases of 5 kg/m^2^ on BMI, increases bladder cancer risk by 3.1% [64,65]. Nonetheless, most metanalyses that support this conclusion have been subject of criticism because of the high heterogeneity between the analysed subgroups of studies [66,67]. Our results suggested that obesity was not associated with an increased risk of bladder cancer.

On the other hand, we found that bladder cancer had a higher incidence in patients with MS (OR 2.8; *p*<.05). Similar results have been reported, suggesting that the components of MS individually carry a risk for bladder cancer [17,68,69]. However, opposite results have been also described; thus further studies are needed to derive more reliable conclusions [70,71].

### Benign prostatic hyperplasia

We found that obesity was associated with an increased incidence of BPH. It has been reported that with each 0.05 increment increase in waist-to-hip ratio there is a 10% increased risk of total (*p*<.003) and severe (*p*<.02) BPH [72]. We also found MS to be associated with an increased risk of BPH, and dyslipidaemia showed the strongest association (OR 2.88; *p*<.05). Patients with MS and BPH have been reported to have a prostatic median annual total growth rate of 1.0 ml/year, while patients with BPH but without MS have a median annual growth rate of 0.64 ml/year (*p*<.05) [73].

## Limitations and strengths

Our study was strengthened by its large sample sizes that drew data from several healthcare organizations across the United States. Furthermore, we were able to use propensity score matching to controls for confounding variables; however, we were not able to adjust the results to all potential confounding variables including smoking status and education level. Our results are additionally strengthened by the ability to assess the chronology of diagnoses and by the wide variety of diagnostic codes that were used. However, our study is limited by the potential for medical coding errors. In the United States the overall misdiagnosis rate is approximately 10–15%, implying that we could have missed or included subjects that did not had the analysed conditions [74]. Moreover, incomplete follow-up, and a lack of granular data to assess causality, which implies that our main outcomes were purely associations, also limited our study. Additionally, our analysis was restrained by the software’s inability to control for more variables while maintaining large sample sizes. We were also limited by the definitions of MS that could be applied to our cohorts.

## Conclusion

In this large cohort of patients, we found that all of the components of MS alone and, especially, in combination, were risk factors for common urological conditions. Obesity showed diverse effects, depending on the disease and BMI-category. The association of MS and ureteral cancer is a novel finding in such a large cohort. These results provide data that can be used in patient counselling about risk of urological diseases with obesity and MS.

These findings also raise the possibility of preventing common urological disease with lifestyle modifications that are an essential components in the management of obesity and MS [75]. Further studies are needed to better understand the potential mechanisms and causality underlying the associations between obesity and MS with common urological conditions. Specifically, large population studies with long follow-up periods and matched case-control models would be valuable in this effort.

## Data Availability

The data that support the findings of this study are available from TriNetX, LLC. Restrictions apply to the availability of these data, which were used under licence for this study. Data are available at https://trinetx.com/products/real-world-datasets/ with the permission of TriNetX, LLC.
